# Automated Volumetric Assessment of the Pallidum and Ventral Diencephalon for Differentiating Progressive Supranuclear Palsy from Parkinson’s Disease

**DOI:** 10.3390/diseases14080271

**Published:** 2026-07-27

**Authors:** Michał Kutyłowski, Piotr Alster, Natalia Madetko-Alster, Bartosz Migda

**Affiliations:** 1 Department of Radiology, Mazovian Brodno Hospital, 03-242 Warsaw, Poland; michael.kutylowski@gmail.com; 2 Department of Neurology, Medical University of Warsaw, 03-242 Warsaw, Poland; piotr.alster@wum.edu.pl (P.A.); natalia.madetko@wum.edu.pl (N.M.-A.); 3 Diagnostic Ultrasound Laboratory, Department of Pediatric Radiology, Medical University of Warsaw, 03-242 Warsaw, Poland

**Keywords:** progressive supranuclear palsy, Parkinson’s disease, pallidum, ventral diencephalon, magnetic resonance imaging

## Abstract

**Background/Objectives:** Progressive supranuclear palsy (PSP) and Parkinson’s disease (PD) share several clinical manifestations, which may complicate differential diagnosis. The aim of this study was to evaluate whether automated volumetric measurements of the pallidum and ventral diencephalon can differentiate PSP from PD. **Methods:** Thirty-two patients were included, comprising 20 patients with PD and 12 patients with PSP. All participants underwent 3-T brain magnetic resonance imaging. Automated segmentation and volumetric analysis were performed using the vol2Brain pipeline. Absolute and normalized volumes of the pallidum and ventral diencephalon were compared between groups. Receiver operating characteristic (ROC) analysis was used to assess diagnostic performance. **Results:** Patients with PSP demonstrated lower pallidal and ventral diencephalic volumes than patients with PD. Differences were observed for both absolute and normalized volumetric measurements (all *p* ≤ 0.002). Total pallidal volume showed the highest diagnostic performance, with an area under the ROC curve (AUC) of 0.958, sensitivity of 85%, and specificity of 100%. Total ventral diencephalon volume also differentiated PSP from PD, yielding an AUC of 0.829, seNsitivity of 70%, and specificity of 92%. **Conclusions:** Pallidal and ventral diencephalic volumes differed between patients with PSP and PD. Automated measurements of pallidal and ventral diencephalic volumes may complement conventional MRI findings in patients with parkinsonian syndromes. Further studies are needed to validate these findings in larger cohorts.

## 1. Introduction

Parkinson’s disease (PD) is the second most common neurodegenerative disease following Alzheimer’s disease. In PD, the neurodegeneration is based on the accumulation of alpha-synuclein in the neurons. The disease is clinically defined by bradykinesia, among other aspects could be mentioned stiffness, resting tremor and response to levodopa treatment [1]. Though the pathology and clinical manifestations are gradually explored, the differentiation at times may seem problematic. This is related to the fact that significant overlaps can be observed in the clinical manifestations between PD and atypical parkinsonisms. A group of atypical parkinsonisms comprises diverse entities including Progressive Supranuclear Palsy (PSP), Corticobasal Degeneration and Multiple System Atrophy. PSP belongs to the group of tauopathies and is the most common atypical parkinsonism [2]. It is defined by the criteria of Hoglinger et al. released in 2017 [3]. Clinical manifestations include akinesia, oculomotor dysfunction, cognitive and language impairment, and postural instability [3]. Due to the clinical overlaps, there is an increased necessity of obtaining efficient supplementary methods of examination. Neuroimaging using magnetic resonance (MR) is the most commonly used tool supporting the differential diagnosis of atypical parkinsonisms. With automated brain volumetry based on MR imaging becoming more widely accessible, the classical radiological signs utilized in diagnostics of PSP (e.g., hummingbird sign [4]) and PD (swallow-tail sign [5]) or even more complex parameters such as midbrain-to-pons ratio and Magnetic Resonance Parkinsonism Index [6] can be complemented by volumetric analysis. Recent advances in automated MRI post-processing have enabled quantitative assessment of regional brain volumes without the need for manual segmentation. Several commercially available and research-oriented platforms provide reproducible volumetric measurements from routine three-dimensional T1-weighted MRI examinations. Such approaches have attracted increasing interest as potential imaging biomarkers for neurodegenerative disorders because they reduce observer variability and facilitate objective assessment of structural brain changes.

Neuropathological and neuroimaging studies indicate that PSP preferentially involves subcortical and mesodiencephalic regions, including the globus pallidus, subthalamic nucleus, substantia nigra, and interconnected basal ganglia pathways [7,8,9,10,11]. Degeneration within these structures contributes to the characteristic motor, postural, and oculomotor manifestations of the disease [8,11]. Automated volumetric analysis enables quantitative assessment of regional brain atrophy and may detect structural changes that are not readily appreciable on routine visual MRI evaluation. Among the structures available in automated segmentation pipelines, the pallidum and ventral diencephalon are of particular interest because they encompass anatomical regions repeatedly implicated in PSP-related neurodegeneration [7,8,9,10,11]. Distinguishing PSP from PD remains difficult, especially during the initial phase of disease when clinical manifestations frequently overlap. As automated MRI volumetry becomes increasingly available in clinical practice, identification of disease-related volumetric changes may improve radiological assessment of patients with suspected PSP.

The aim of this study was to evaluate whether automated volumetric measurements of the pallidum and ventral diencephalon differ between patients with PSP and PD and to assess their diagnostic performance. We hypothesized that both structures would demonstrate lower volumes in PSP, reflecting the preferential involvement of subcortical and mesodiencephalic regions in this disorder. These structures were selected a priori because they are consistently involved in PSP neuropathology and can be reliably segmented using the vol2Brain pipeline.

## 2. Materials and Methods

### 2.1. Participants

The study enrolled 32 patients (20 with PD and 12 with PSP) examined from 2024 to 2026, with the diagnosis based on clinical criteria and established by an experienced neurologist. Parkinson’s disease was diagnosed according to the Movement Disorder Society clinical diagnostic criteria [1], whereas PSP was diagnosed according to the 2017 Movement Disorder Society PSP criteria [3]. Given that most of the patients are still alive, the histopathological verification was not carried out. The approximate mean duration of the disease at the time of magnetic resonance imaging (MRI) acquisition was 3 years for patients with PSP and 1.5 years for patients with PD. The following exclusion criteria were established: severe small vessel disease (defined as Fazekas 2 and 3) [12], significant areas of encephalomalacia (particularly involving deep brain structures), and the presence of an intra-axial tumor. None of the patients met any of the exclusion criteria. The study was conducted in accordance with the 1964 Helsinki declaration. Participants gave written, informed consent. The study was approved by the Ethical Committee of the Medical University of Warsaw (approval no. AKBE243/2016).

### 2.2. Magnetic Resonance Imaging

All of the enrolled patients underwent magnetic resonance imaging of the brain performed on a 3.0-Tesla MR scanner MAGNETOM Skyra, (Siemens Healthineers, Erlangen, Germany) equipped with a standard head coil. The 3D T1-weighted Magnetization Prepared Rapid Gradient Echo (MPRAGE) sequence with an isotropic spatial resolution of 1 mm × 1 mm × 1 mm was included in the protocol to support optimal resolution for further volumetric analysis [13].

### 2.3. MRI Analysis and Volumetry

**Figure 1:**
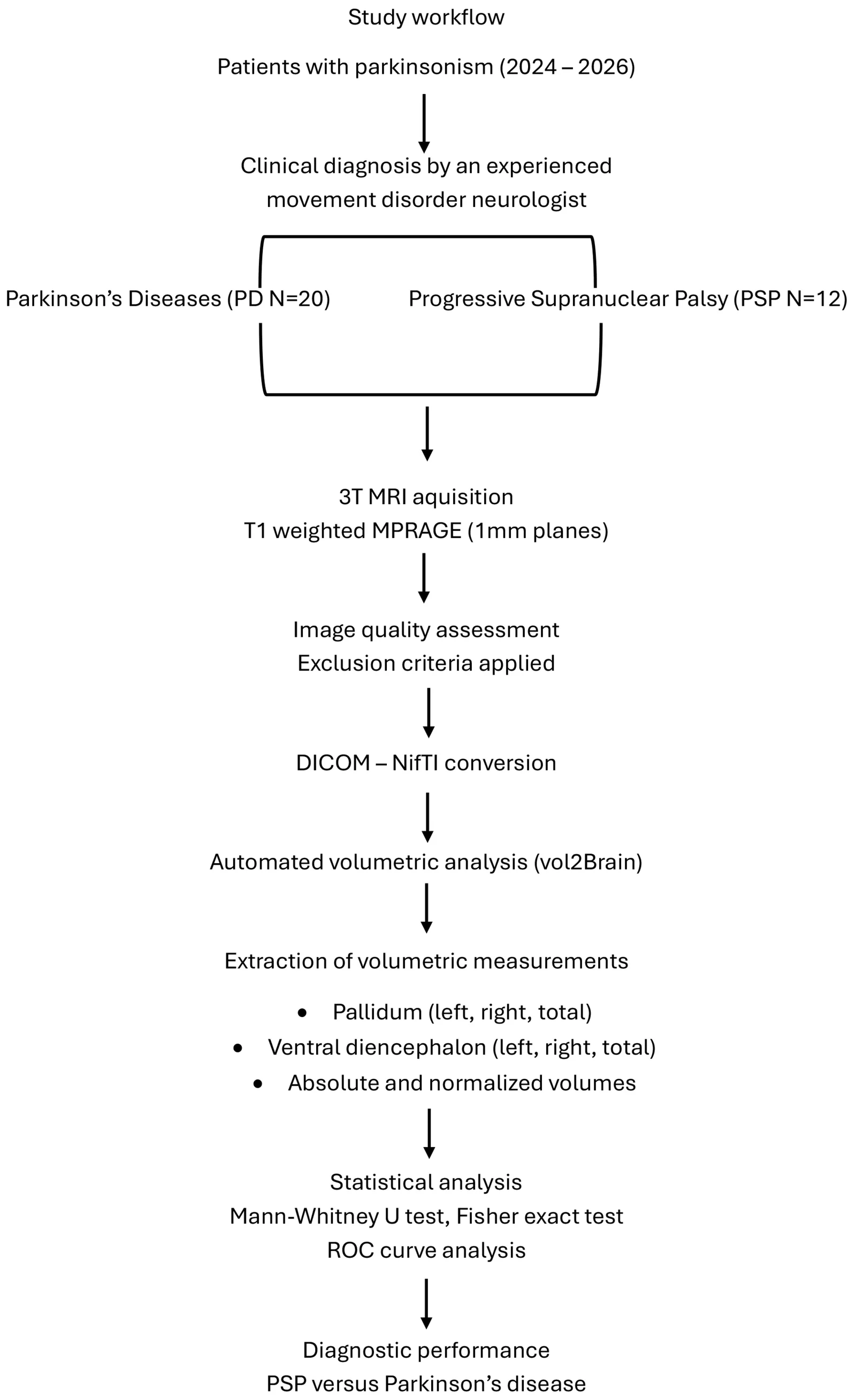
Study workflow.

All obtained MR images were initially reviewed for quality, exclusion criteria and structural abnormalities by a board-certified radiologist with more than 5 years of experience in neuroimaging. Subsequently the examinations were anonymized and converted from standard DICOM format into NIfTI format utilizing the MRIcroGL application (obtained from www.nitrc.org accessed on 8 June 2026). Once converted, the NIfTI files were submitted to the volBrain online system to proceed with an automatic volumetric analysis with the vol2Brain pipeline [14,15]. The vol2Brain pipeline performs automated preprocessing, including intensity normalization, tissue classification, atlas-based segmentation, and volumetric estimation. Following image upload, all processing steps were completed automatically without manual segmentation or user-guided editing. All segmentations were visually inspected to exclude processing failures before statistical analysis. The diagnostic performance of the vol2Brain segmentation algorithm has been validated previously against expert manual segmentations; therefore, no additional segmentation accuracy analysis was performed in the present study [14,15]. The brain structures labeling was based on the Neuromorphometrics atlas definitions (Neuromorphometrics, Inc. Somerville, MA, USA, http://neuromorphometrics.com, accessed on 8 June 2026). Obtained volumetric measurements were exported for further statistical analysis. No custom software or computer code was developed for this study. All volumetric analyses were performed using the publicly available vol2Brain pipeline. The overall study workflow is presented in Figure 1.

### 2.4. Statistical Analysis

All statistical analyses were performed using STATISTICA software (version 13.1; TIBCO Software Inc., Palo Alto, CA, USA). The distribution of continuous variables was assessed using the Shapiro–Wilk test. Continuous variables were presented as median and interquartile range (IQR), whereas categorical variables were expressed as counts and percentages.

Comparisons between patients with Parkinson’s disease and progressive supranuclear palsy were performed using the Mann–Whitney U test for continuous variables and Fisher’s exact test for categorical variables. All statistical tests were two-tailed, and statistical significance was set at *p* < 0.05.

Receiver operating characteristic (ROC) analysis was performed to evaluate the diagnostic performance of selected volumetric parameters. The area under the curve (AUC), 95% confidence intervals (CI), sensitivity (Se), and specificity (Sp) were calculated. Optimal cut-off values were determined using the Youden index.

## 3. Results

### 3.1. Study Population

A total of 32 patients were included in the study, comprising 20 patients with Parkinson’s disease (PD) and 12 patients with progressive supranuclear palsy (PSP). The groups did not differ significantly in age (72.0 [67.75–73.25] vs. 67.5 [65.5–72.5] years, *p* = 0.234) or sex distribution (65.0% vs. 58.3% male, *p* = 0.724), indicating comparable demographic characteristics (Table 1).

### 3.2. Volumetric Analysis

**Figure 2:**
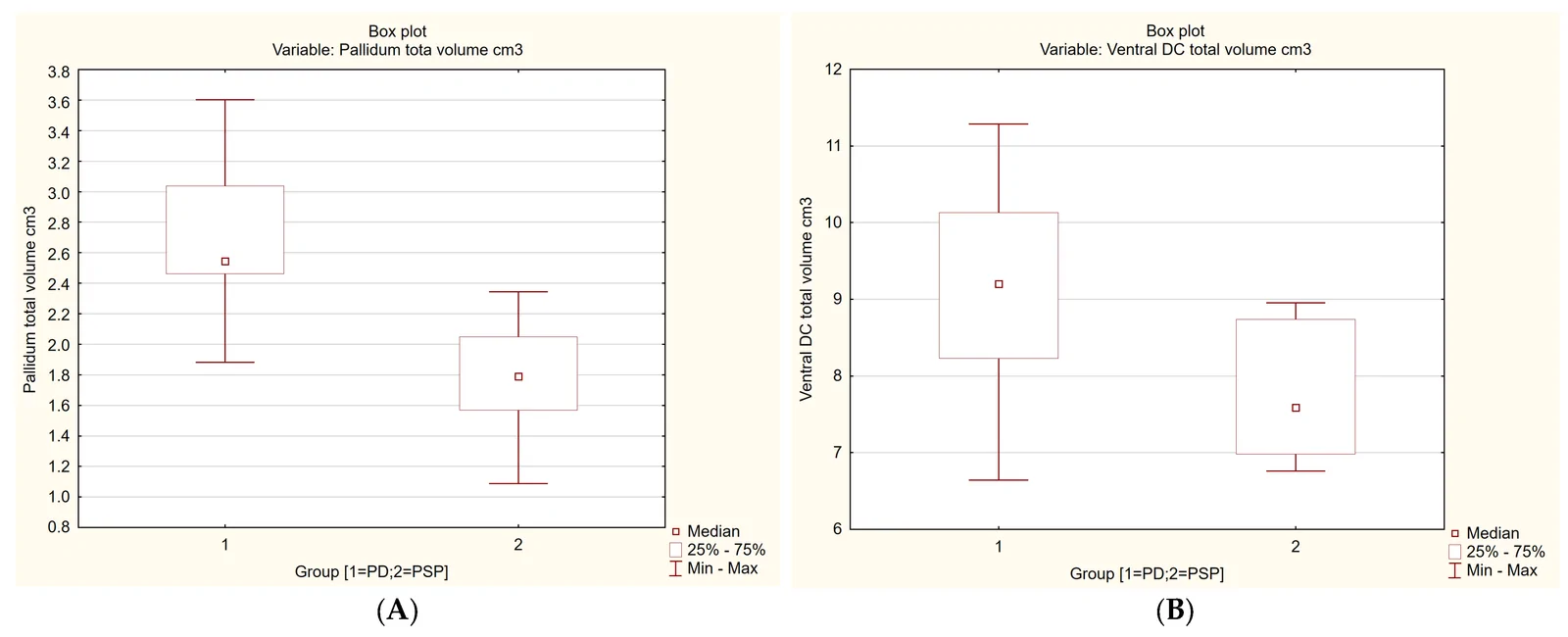
Distribution of total pallidal (**A**) and ventral diencephalic (**B**) volumes in patients with Parkinson’s disease and progressive supranuclear palsy. Squares indicate medians, boxes represent interquartile ranges, and whiskers indicate minimum and maximum values.

**Figure 3:**
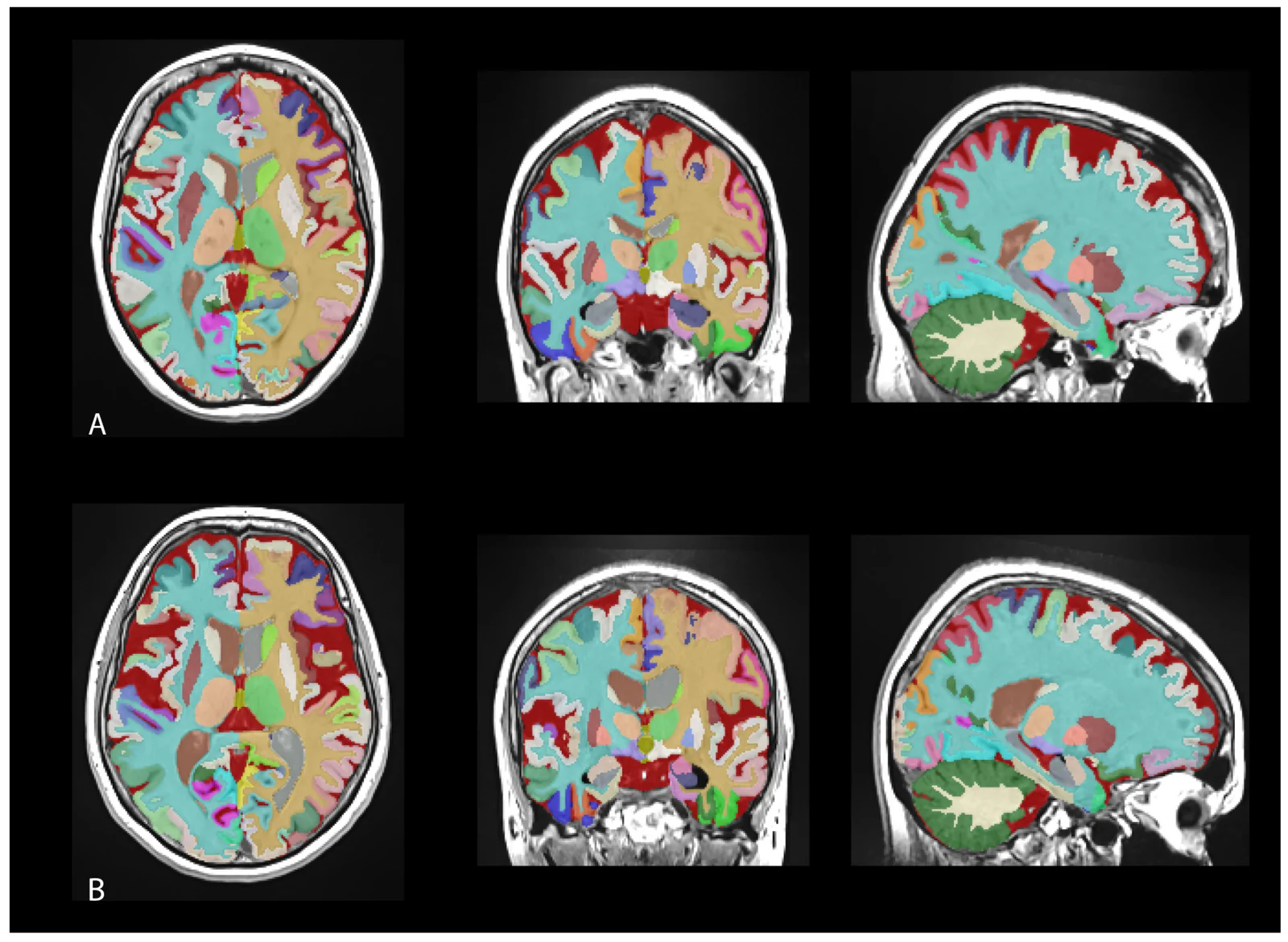
Representative automated vol2Brain segmentation in Parkinson’s disease (**A**) and progressive supranuclear palsy (**B**). Representative axial, coronal, and sagittal T1-weighted MR images with automated segmentation generated using the vol2Brain pipeline. Panel (**A**) shows a patient with Parkinson’s disease (PD), whereas panel (**B**) shows a patient with progressive supranuclear palsy (PSP). The PSP case demonstrates reduced pallidal and ventral diencephalic volumes compared with the representative PD case, consistent with the quantitative volumetric findings presented in Table 2. For the illustrated cases, total pallidal volume was 2.48 cm^3^ (0.201%) in PD versus 1.58 cm^3^ (0.120%) in PSP, while total ventral diencephalon volume was 8.93 cm^3^ (0.725%) versus 7.21 cm^3^ (0.551%), respectively.

Absolute pallidal and ventral diencephalic volumes were lower in the PSP group than in the PD group. The largest differences were observed for total ventral diencephalon volume (9.20 [8.26–10.11] vs. 7.59 [7.01–8.69] cm^3^, *p* = 0.002) and total pallidal volume (2.54 [2.46–3.00] vs. 1.79 [1.57–2.05] cm^3^, *p* < 0.001). Similar differences were observed for the left and right hemispheres (Table 2). The distribution of total pallidal and ventral diencephalic volumes in both groups is presented in Figure 2A,B. Representative examples of automated segmentation obtained using the vol2Brain pipeline are shown in Figure 3.

When normalized to total brain volume, both structures remained significantly smaller in PSP. Total ventral diencephalon volume accounted for 0.643% (IQR 0.598–0.654) of total brain volume in PD and 0.542% (IQR 0.527–0.573) in PSP (*p* < 0.001). Likewise, total pallidal volume represented 0.180% (IQR 0.161–0.200) of total brain volume in PD and 0.127% (IQR 0.113–0.149) in PSP (*p* < 0.001) (Table 2).

### 3.3. ROC Curve Analysis

**Figure 4:**
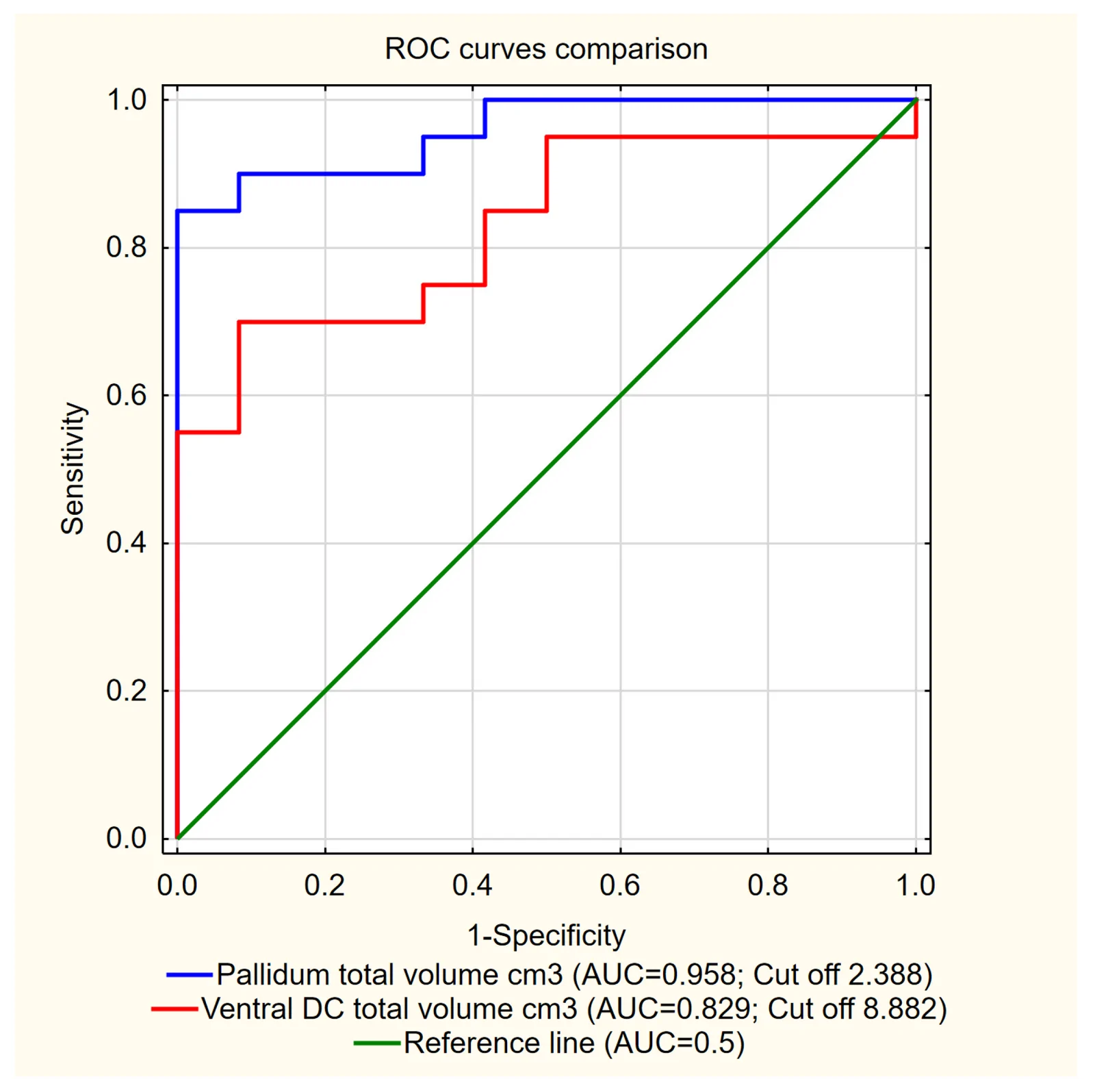
Receiver operating characteristic (ROC) curves for total pallidal volume and total ventral diencephalon volume differentiating progressive supranuclear palsy from Parkinson’s disease.

Total pallidal volume demonstrated the highest diagnostic performance for differentiating PSP from PD, with an AUC of 0.958 (95% CI 0.897–1.000), sensitivity of 85%, and specificity of 100%. Total ventral diencephalon volume also showed good discriminatory ability, achieving an AUC of 0.829 (95% CI 0.687–0.971), sensitivity of 70%, and specificity of 92% (Table 3). ROC curves for both volumetric parameters are presented in Figure 4. Both volumetric parameters demonstrated statistically significant discriminatory ability. Total pallidal volume provided higher specificity and overall diagnostic accuracy than ventral diencephalon volume.

## 4. Discussion

In the present cohort, patients with PSP exhibited smaller pallidal and ventral diencephalic volumes than patients with PD. Both structures showed good discriminatory performance, with total pallidal volume demonstrating the highest diagnostic accuracy. Our results indicate that automated volumetric assessment of selected subcortical regions may serve as an additional imaging marker in the differential diagnosis of parkinsonian syndromes. Objective volumetric measurements may be particularly useful in patients presenting with early or clinically equivocal parkinsonian syndromes, in whom conventional MRI findings remain inconclusive. Because volumetry can be derived from routine three-dimensional MRI acquisitions, it may be readily incorporated into existing imaging workflows.

The observed pallidal atrophy is consistent with current knowledge regarding the neuropathology of PSP. Neuropathological studies indicate that tau pathology in PSP affects multiple interconnected subcortical structures rather than the brainstem alone, including the globus pallidus, subthalamic nucleus and substantia nigra. Neuropathological studies have demonstrated marked neuronal loss and gliosis within these structures, supporting their central role in disease pathogenesis [8]. In vivo imaging studies have similarly shown preferential involvement of subcortical nuclei in PSP, with structural changes extending beyond the classical midbrain abnormalities traditionally emphasized in routine MRI assessment [7]. More recently, longitudinal imaging studies have shown progressive involvement of subcortical and mesencephalic structures across different PSP phenotypes, indicating that these changes represent a dynamic component of disease progression rather than isolated anatomical abnormalities [9]. These pathological changes precede advanced neuronal loss and are reflected by progressive regional atrophy detectable with structural MRI.

The diagnostic significance of ventral diencephalon volume is less straightforward because this label encompasses multiple anatomically distinct structures. At present, the current version of the vol2Brain pipeline does not provide reliable segmentation of individual components of this anatomical region. Consequently, the observed volumetric changes should be interpreted as reflecting the entire ventral diencephalic compartment rather than any single structure. Within the Neuromorphometrics atlas used by vol2Brain, the ventral diencephalon includes the subthalamic region, hypothalamic structures, substantia nigra, and adjacent mesodiencephalic pathways [13,14,15]. Several of these regions are known to be involved early in PSP and contribute to characteristic motor, postural, and oculomotor manifestations of the disease [11]. Therefore, the observed reduction in ventral diencephalon volume probably represents combined tissue loss within several neighboring mesodiencephalic structures rather than selective atrophy of a single nucleus.

This interpretation is supported by recent MRI-based staging studies. Planche et al. demonstrated that PSP-related atrophy follows a characteristic spatial progression involving interconnected basal ganglia, diencephalic, and brainstem regions [10]. Similarly, Kumar et al. reported distinct but overlapping trajectories of regional brain atrophy among different PSP phenotypes [9,10]. The lower pallidal and ventral diencephalic volumes observed in the present study are consistent with these network-based models of neurodegeneration.

The observed diagnostic performance indicates that volumetric measurements of the pallidum and ventral diencephalon may aid differentiation between PSP and PD. Total pallidal volume achieved an AUC of 0.958 with 100% specificity, while total ventral diencephalon volume achieved an AUC of 0.829. Although these findings should be interpreted cautiously because of the limited sample size, they suggest that automated volumetric measurements may capture disease-related structural changes relevant to the differential diagnosis of PSP and PD.

At present, the most widely validated MRI biomarkers of PSP remain the midbrain-to-pons ratio, MRPI, and MRPI 2.0 [16,17]. Quattrone et al. demonstrated excellent diagnostic performance of MRPI-based approaches for differentiating PSP from PD, including patients with PSP-parkinsonism [17]. Similarly, Madetko et al. reported that MRPI 2.0 may improve diagnostic accuracy in selected clinical scenarios involving atypical parkinsonian syndromes [16]. The present findings should therefore be regarded as complementary to these established imaging markers rather than as an alternative to them. Future multimodal MRI approaches combining morphometric indices with automated volumetry may further improve diagnostic performance and deserve prospective evaluation. One practical advantage of automated volumetry is that it can be obtained directly from routine three-dimensional T1-weighted MRI acquisitions with limited operator dependency. This approach is further facilitated by the increasing availability of automated volumetric platforms such as volBrain and vol2Brain [14,15].

An unexpected finding was that relative volumetric measures expressed as a percentage of total brain volume were lower in PSP and followed the same direction as the absolute measurements. This observation suggests that the detected differences were not solely attributable to global brain volume variation and supports the presence of regional structural involvement affecting the pallidum and ventral diencephalon in PSP.

Several limitations should be acknowledged. First, ventral diencephalon represents a composite anatomical label, preventing identification of the specific structures responsible for the observed differences. Second, automated segmentation of mesodiencephalic regions remains technically challenging, particularly when boundaries between adjacent nuclei are poorly defined on conventional MRI. Third, the relatively small sample size limits the generalizability of the findings. Furthermore, the PSP cohort was not stratified according to clinical phenotype. Previous studies have demonstrated that different PSP variants may exhibit distinct patterns and rates of regional atrophy, which could influence volumetric measurements and diagnostic performance [9]. Detailed clinical measures, including MDS-UPDRS scores, Hoehn and Yahr stage, levodopa responsiveness, and treatment status, were not consistently available because of the retrospective design of the study. Finally, diagnoses were established clinically without neuropathological confirmation. Although the MDS PSP criteria provide high diagnostic accuracy, clinical misclassification remains possible, particularly during the early stages of disease [3].

Future studies should determine whether these findings can be reproduced in larger multicentre cohorts and investigate whether similar volumetric patterns are observed across different PSP phenotypes. More detailed segmentation of mesodiencephalic structures may help identify the anatomical substrates responsible for the observed reductions in ventral diencephalon volume. It would also be valuable to compare results obtained using different volumetric pipelines and to assess whether combining volumetric parameters with established MRI biomarkers such as MRPI or MRPI 2.0 improves diagnostic performance [16,17]. Future studies should also evaluate multimodal biomarker models integrating MRI volumetry with clinical, biochemical, and advanced neuroimaging markers and also include standardized clinical severity scales, including MDS-UPDRS and Hoehn&Yahr staging.

## 5. Conclusions

Patients with progressive supranuclear palsy showed lower pallidal and ventral diencephalic volumes than patients with Parkinson’s disease. Both parameters differentiated the study groups and may complement conventional MRI assessment in the evaluation of parkinsonian syndromes. Confirmation in larger prospective multicentre studies is required before routine clinical implementation.

## Figures and Tables

**Table 1:** Demographic characteristics of the study population.

Variable	Total (N = 32)	PD (N = 20)	PSP (N = 12)	*p*-Value
Age, years	71.5 (66.0–73.25)	72.0 (67.75–73.25)	67.5 (65.5–72.5)	0.234 *
Male sex, *n* (%)	20 (62.5%)	13 (65.0%)	7 (58.3%)	0.724 **
Female sex, *n* (%)	12 (37.5%)	7 (35.0%)	5 (41.7%)	—

Legend: *p*-value—* Mann–Whitney U test, ** Fisher exact test.

**Table 2:** Comparison of absolute (cm^3^) and normalized (% of total brain volume) pallidal and ventral diencephalic volumes in patients with Parkinson’s disease and progressive supranuclear palsy.

Parameter	PD (N = 20) Median (Q1–Q3)	PSP (N = 12) Median (Q1–Q3)	*p*-Value
Ventral DC left volume (cm^3^)	4.60 (4.21–5.11)	3.83 (3.61–4.42)	0.006
Ventral DC right volume (cm^3^)	4.60 (4.08–5.02)	3.78 (3.45–4.25)	0.001
Ventral DC total volume (cm^3^)	9.20 (8.26–10.11)	7.59 (7.01–8.69)	0.002
Ventral DC left volume (%)	0.322 (0.298–0.331)	0.279 (0.270–0.288)	0.001
Ventral DC right volume (%)	0.315 (0.300–0.329)	0.266 (0.259–0.284)	<0.001
Ventral DC total volume (%)	0.643 (0.598–0.654)	0.542 (0.527–0.573)	<0.001
Pallidum left volume (cm^3^)	1.28 (1.22–1.57)	0.89 (0.81–1.04)	<0.001
Pallidum right volume (cm^3^)	1.30 (1.21–1.48)	0.91 (0.73–1.02)	<0.001
Pallidum total volume (cm^3^)	2.54 (2.46–3.00)	1.79 (1.57–2.05)	<0.001
Pallidum left volume (%)	0.092 (0.081–0.102)	0.065 (0.058–0.074)	<0.001
Pallidum right volume (%)	0.091 (0.085–0.096)	0.064 (0.051–0.072)	<0.001
Pallidum total volume (%)	0.180 (0.161–0.200)	0.127 (0.113–0.149)	<0.001

Legend: Data are presented as median (IQR). *p*-values were obtained using the Mann–Whitney U test. Percentages represent the proportion of total brain volume.

**Table 3:** Diagnostic performance of volumetric parameters for differentiating PSP from PD.

Variable	AUC	95% CI	*p*	Cut Off	Se	Sp
Pallidum total volume cm^3^	0.958	0.897–1.0	<0.001	2.388	0.85	1.00
Ventral DC total volume cm^3^	0.829	0.687–0.971	<0.001	8.882	0.70	0.92

Legend: AUC—area under the ROC curve; CI—confidence interval; *p*—*p*-value for counted AUC; Se—sensitivity. Sp—specificity.

## Data Availability

The data that support the findings of this study are not publicly available because they contain potentially identifiable participant information. De-identified data may be available from the corresponding author on reasonable request, subject to ethics approval and institutional requirements.
